# Uncovering the Potential Mechanisms of Ergothioneine in Neuroinflammation Through Network Pharmacology, Molecular Docking, Molecular Dynamics Simulation, and In Vitro Validation

**DOI:** 10.3390/ijms27052179

**Published:** 2026-02-26

**Authors:** Deyou Cao, Jingxuan Jia, Yishu Yin, Weihong Lu

**Affiliations:** 1 School of Medicine and Health, Faculty of Life Science and Medicine, Harbin Institute of Technology, Harbin 150001, China; 17703601666@163.com; 2 Zhengzhou Advanced Research Institute, Harbin Institute of Technology, Zhengzhou 450000, China; 3 School of Chemistry and Chemical Engineering, Harbin Institute of Technology, Harbin 150001, China

**Keywords:** neuroinflammation, ergothioneine, network pharmacology, computational analysis, PI3K/AKT signaling pathway, NF-κB signaling pathway

## Abstract

Neuroinflammation is a critical pathological process implicated in several neurological disorders. It arises from complex interactions among immune cells and the excessive release of pro-inflammatory mediators, ultimately leading to neuronal damage. Ergothioneine (EGT), a naturally occurring antioxidant, has attracted attention for its potential anti-inflammatory role in neuroinflammation, although it remains poorly understood. We employed a comprehensive strategy combining network pharmacology, molecular docking, molecular dynamics simulations, and in vitro experiments to explore how EGT influences neuroinflammatory pathways. Computational analyses indicated that EGT might regulate several inflammation-related signaling cascades by targeting key molecules such as Tumor Necrosis Factor (TNF), AKT Serine/Threonine Kinase 1 (AKT1), Caspase 3 (CASP3), and Interleukin 6 (IL-6). Docking and dynamics simulations confirmed strong and stable binding between EGT and these targets. Experiments using lipopolysaccharide-stimulated BV2 microglia cells demonstrated that EGT significantly reduced pro-inflammatory cytokine production, primarily through modulation of the phosphoinositide 3-kinase (PI3K)/protein kinase B (AKT) and nuclear factor kappa-light-chain-enhancer of activated B cells (NF-κB) signaling pathways. By integrating multi-omics approaches with cellular validation, this study sheds light on the molecular mechanisms underlying EGT’s anti-inflammatory effect and supports its potential application as a functional food ingredient for managing neuroinflammation.

## 1. Introduction

Neuroinflammation plays a pivotal role in the development of many neurological disorders and has become a major focus in neuroscience research. This process is triggered by diverse pathological stimuli and involves complex interactions among immune cells, neurons, and glial cells [1]. Microglia, the innate immune cells of the central nervous system, are among the first to respond to injury signals [2]. When activated, they transition from a resting, branched morphology to an amoeboid shape and release large amounts of pro-inflammatory cytokines, chemokines, and reactive oxygen species [3]. These mediators have dual effects: they attract peripheral immune cells into the central nervous system, amplifying inflammation, while simultaneously causing direct neuronal damage [4]. For example, Tumor Necrosis Factor-alpha (TNF-α) activates apoptotic pathways by binding to neuronal receptors, ultimately promoting neuronal death [5]. Similarly, Interleukin-1 beta (IL-1β) interferes with neurotransmitter synthesis and release, impairing neuronal signaling [6].

Ergothioneine (EGT) is a naturally occurring betaine-type amino acid found in bacteria, plants, and animals. Mushrooms have been identified as major natural sources of EGT [7], with *Pleurotus citrinopileatus* Singer varieties containing markedly higher levels than most other edible species. EGT has recently been approved by the European Food Safety Authority and recognized as safe by the U.S. Food and Drug Administration [8,9,10]. Studies suggest that EGT possesses antioxidant, anti-inflammatory, and cytoprotective properties and may also contribute to healthy aging and cardiovascular disease prevention [11,12,13]. Its safety, unique antioxidant capacity, targeted anti-inflammatory effects, anti-aging benefits, non-competitive whitening properties, and excellent stability have also made it a popular ingredient in skincare formulations [9].

With growing recognition of the complexity of disease mechanisms, traditional single-target strategies have been shown to be insufficient for elucidating disease networks or drug action pathways. Network pharmacology, an emerging systems-based approach, has helped uncover regulatory mechanisms and drug–target interactions at a network-wide level, providing systematic frameworks for mechanism discovery [14,15]. Molecular docking stimulates geometric and energetic matching to predict molecular recognition events, offering a theoretical foundation for forecasting drug activity and clarifying mechanisms of action. Molecular dynamic (MD) complements docking by numerically solving motion equations to dynamically study molecular structures and properties [16]. The neuroprotective effects of EGT have attracted widespread attention in recent years. MD simulations have demonstrated that EGT directly interacts with α-synuclein pentamers, disrupting their structural stability and thereby preventing protein aggregation in Parkinson’s disease models [17]. Similarly, network pharmacology, molecular docking, and MD simulations have been successfully applied to structurally related antioxidants (e.g., ferulic acid) to elucidate their inhibitory effects on neuroinflammatory enzymes such as monoamine oxidase B [18]. When integrated with pharmacophore modeling, these techniques offer comprehensive support for multidimensional analysis of EGT mechanisms.

While the neuroprotective efficacy of EGT is well documented, a comprehensive understanding of its systemic molecular mechanism remains elusive. The existing literature largely emphasizes macro-level phenotypic changes, leaving a gap in our knowledge regarding target-specific binding kinetics and dynamic stability. To bridge this gap, this study employs a synergistic tripartite framework: (1) utilizing network pharmacology to map the interconnected target network; (2) performing MD simulations to evaluate the thermodynamic stability of EGT within candidate protein pockets; and (3) implementing in vitro assays to confirm the modulation effect on key neuroinflammatory signaling pathways. This integrated pipeline moves beyond the limitations of conventional single-dimensional studies, offering a more precise resolution of EGT’s bioactivity and reinforcing its potential as a high-value active compound in the functional food industry.

## 2. Results

### 2.1. Targeting Ergothioneine in the Treatment of Neuroinflammation

**Figure 1:**
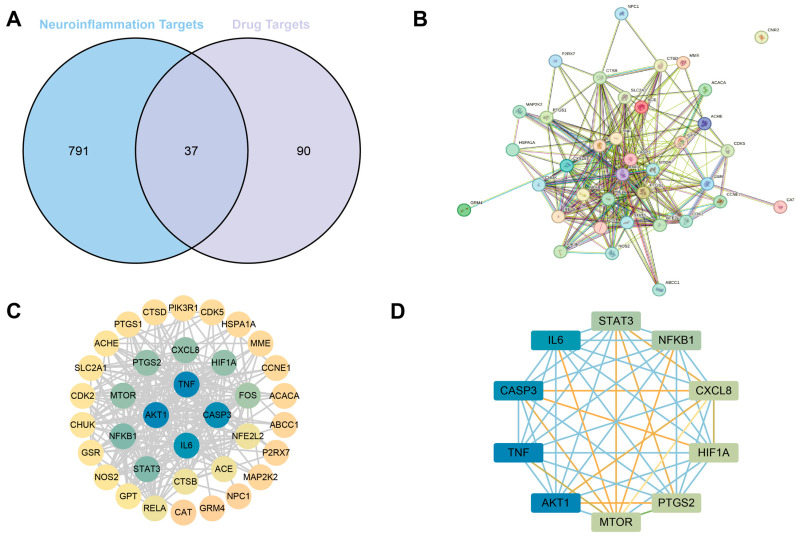
Target prediction of EGT neuroinflammation improvement. (**A**) Venn diagram analysis of EGT’s neuroinflammation targets; (**B**) PPI network analysis of EGT neuroinflammation targets; (**C**) ranking of EGT neuroinflammation targets by severity; (**D**) top 10 EGT neuroinflammation targets.

We identified 37 overlapping targets between EGT-associated genes and neuroinflammation-related genes (Figure 1A). These shared targets suggest that EGT may modulate neuroinflammation through multiple mechanisms involving these 37 candidate genes (Supplementary Table S1). The PPI analysis revealed that TNF, AKT1, CASP3, and IL-6 are highly connected hub nodes, suggesting that these molecules likely serve as core targets through which EGT exerts its anti-inflammatory effects (Figure 1C,D). Peripheral proteins such as NFKB1, STAT3, and MTOR interact with these core targets, forming a complex regulatory network. The overall network density supports the notion that EGT operates through multi-target and multi-pathway mechanisms rather than a single-target mode of action. Based on the Degree value, a refined subnetwork was formed to highlight the most influential nodes (Figure 1D). The core targets, IL-6, CASP3, TNF, and AKT1, were found to be closely associated with proteins involved in inflammatory and signaling pathways, such as STAT3, NFKB1, CXCL8, HIF1A, MTOR, and PTGS2. This network indicates that EGT exerts its effects through multiple mechanisms, regulating inflammatory mediators (TNF, IL-6, CXCL8), apoptosis-related proteins (CASP3), and signaling cascades (AKT1/STAT3/NFKB1/MTOR).

### 2.2. Biological Mechanism of Ergothioneine in Neuroinflammation

**Figure 2:**
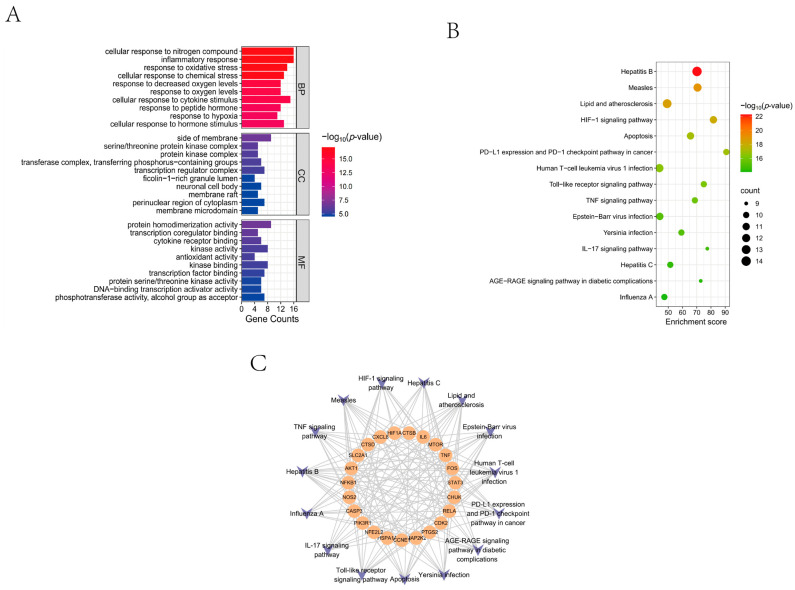
Analysis of EGT neuroinflammatory target profiles. (**A**) GO analysis of EGT neuroinflammatory targets; (**B**) KEGG pathway analysis of EGT neuroinflammatory targets; (**C**) enrichment pathway–target network diagram. Circular nodes represent targets; irregular nodes represent signaling pathways. Lines indicate enrichment relationships between targets and pathways.

Gene Ontology (GO) and Kyoto Encyclopedia of Genes and Genomes (KEGG) enrichment analyses were performed on 37 overlapping targets using medascape (Figure 2A). GO classification categorized genes into Biological Process (BP), Cellular Component (CC), and Molecular Function (MF). BP analysis indicated enrichment in inflammatory responses, cytokine signaling, and LPS-related processes, suggesting EGT’s potential role in modulating immune–inflammatory pathways. CC terms were enriched in kinase complexes, transcriptional regulation assemblies, and postsynaptic compartments, indicating involvement in signal transduction and neuronal activity. MF analysis emphasized kinase activity and transcriptional co-regulator binding, demonstrating the importance of these targets’ phosphorylation cascades and transcriptional control. Overall, these findings highlighted strong links between the targets and processes central to inflammation, immune regulation, and synaptic function.

KEGG pathway analysis identified key pathways, including Toll-like receptor, TNF, Interleukin-17 (IL-17), apoptosis, and Hypoxia-inducible factor-1 (HIF-1) signaling, all of which are closely related to immune response and cell death (Figure 2B). The pathways–target interaction network (Figure 2C) showed that core targets such as HIF1A, IL-6, mTOR, and TNF participate in multiple interconnected pathways, reinforcing their role in coordinating inflammatory and metabolic responses.

### 2.3. Molecular Docking of EGT with Key Targets

To evaluate direct molecular interactions, docking studies were conducted between EGT and four key proteins, AKT1, CASP3, IL-6, and TNF, using Cb-Dock2. The results indicate that EGT exhibited the strongest affinity for AKT1, moderate binding to TNF, and weaker interactions with CASP3 and IL-6 (Table 1), suggesting that its primary modulation lies in cell survival and inflammatory signaling, with a lesser direct impact on apoptosis or cytokine regulation.

**Figure 3:**
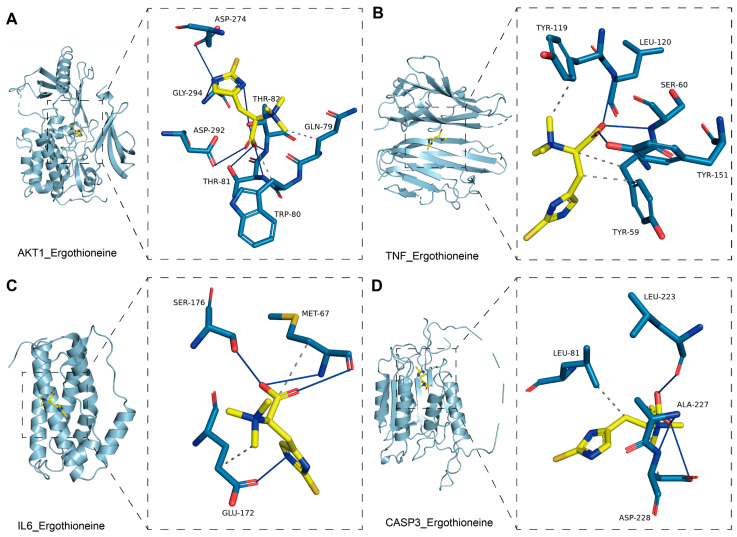
Molecular docking visualization of ligand–protein interactions. Molecular docking analysis demonstrates that residues including AKT1, CASP3, IL-6 and TNF significantly contribute to binding affinity. (**A**) Molecular docking of EGT with AKT1. (**B**) Molecular docking of EGT with CASP3. (**C**) Molecular docking of EGT with IL-6. (**D**) Molecular docking of EGT with TNF. Note: Blue solid lines indicate hydrogen bonds, and gray dashed lines represent hydrophobic interactions.

Further analysis using Protein-Ligand Interaction Profiler (PLIP) and PyMOL Molecular Graphics System (PyMOL) provided detailed interaction profiles and 3D structural models. Figure 3A,B illustrate that EGT forms stable complexes with AKT1 and TNF, supported by multiple hydrogen bonds and extensive hydrophobic contacts, which enhance ligand–receptor affinity and suggest a strong modulatory effect on AKT1-mediated signaling and TNF-associated inflammation. In contrast, Figure 3C,D show that CASP3 and IL-6 interactions involve fewer hydrogen bonds and limited hydrophobic contacts, indicating weaker binding stability. This implies that the direct regulatory influence of EGT on CASP3 and IL-6 is likely modest, suggesting more indirect or secondary roles in apoptosis and cytokine regulation compared with its interactions with AKT1 and TNF. Overall, these findings highlight distinct binding behaviors of EGT across different target proteins and support its involvement in neuroinflammation through a multi-target, multi-level interaction mechanism.

### 2.4. Molecular Dynamics Simulations Reveal the Binding Mechanism of EGT

**Figure 4:**
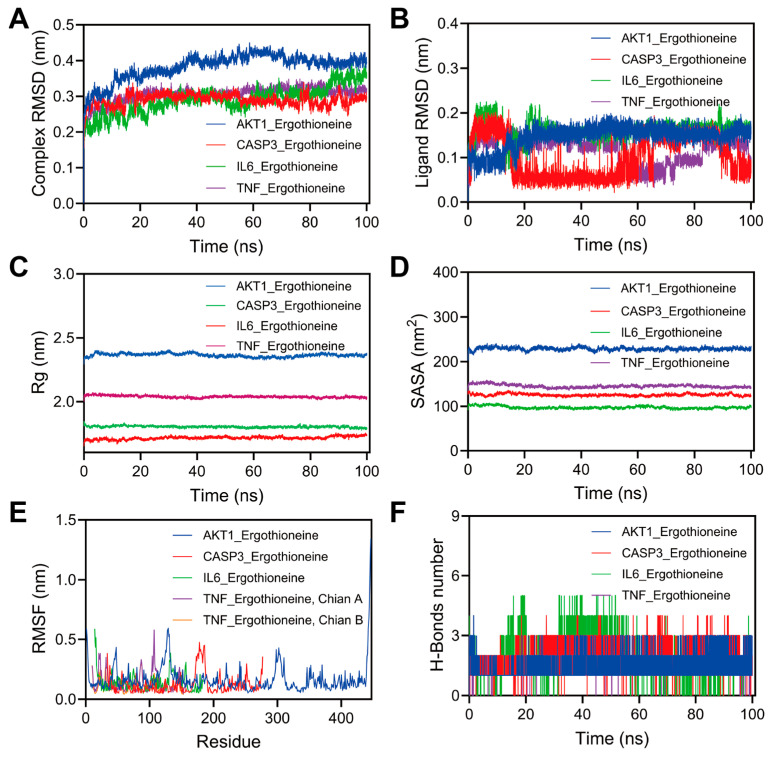
Molecular dynamics simulation results demonstrating stability and conformational changes of ligand–protein complexes over 100 ns simulation. (**A**) Protein–ligand complex RMSD, (**B**) ligand RMSD, (**C**) Rg curve, (**D**) SASA curve, (**E**) RMSF curve, and (**F**) hydrogen bond change curve.

Guided by the molecular docking results, we carried out 100 ns MD simulations for the four protein–ligand complexes and evaluated their stability and conformational behavior using root mean square deviation (RMSD), root mean square fluctuation (RMSF), radius of gyration (Rg), and solvent-accessible surface area (SASA) (Figure 4).

RMSD is a key measure for evaluating the structural stability of protein–ligand complexes during stimulation [19]. LowerRMSD values generally indicate stronger and more stable interactions [20]. In this study, TNF_EGT demonstrated excellent stability, whereas AKT1_EGT showed a slightly higher RMSD of approximately 0.4 nm, indicating minor structural adjustments that remain within acceptable limits (Figure 4A).

As shown in Figure 4B, the ligand RMSD values in the AKT1_EGT and IL6_EGT systems rapidly stabilize after a brief equilibration period (approximately 0.12–0.18 nm), indicating that ergothioneine adopts a stable conformation within the binding pocket and maintains a consistent binding mode. In contrast, the CASP3_EGT and TNF_EGT systems exhibit markedly larger ligand RMSD fluctuations, suggesting higher conformational flexibility and more frequent binding rearrangements of the binding pose. These dynamics reflect relatively weaker protein–ligand interaction stability in the CASP3_EGT and TNF_EGT complexes. Among the systems analyzed, AKT1_EGT displayed the lowest and most stable ligand RMSD, suggesting a compact and conformationally constrained binding environment. The Rg value of AKT1_EGT is slightly higher, likely due to its more complex protein structure (Figure 4C). Larger SASA values indicate more extensive solvent exposure, while smaller values correspond to more compact folding and decreased thermal susceptibility. Across complexes, SASA values remained relatively stable, suggesting no significant unfolding. AKT1–EGT showed the highest SASA, consistent with its larger surface area (Figure 4D). AKT1–EGT exhibited elevated fluctuations in specific regions (e.g., N-terminal or loop domains), suggesting higher flexibility during ligand binding, whereas CASP3–EGT showed lower fluctuations, indicating more rigid binding states (Figure 4E).

Furthermore, hydrogen bonds (H-bonds), as a strong non-covalent interaction, facilitate interactions between atoms on the protein surfaces and solvent molecules and help the ligand recognize precise interactions with the active site [21,22]. Therefore, the number of hydrogen bonds is also used to determine the stability of protein–ligand complexes during simulation. To further investigate hydrogen bond variations in the complex system, we conducted statistical analysis of intermolecular hydrogen bonds formed by small-molecule proteins during the 100 ns molecular simulation. As shown in Figure 4F, AKT1_EGT formed numerous hydrogen bonds (maintaining 1–4 bonds) throughout the simulation, indicating strong binding stability with AKT1. TNF_EGT also maintained a moderate number of hydrogen bonds. In contrast, the CASP3-IL-6 complex exhibited fewer hydrogen bonds, suggesting that its binding stability primarily relies on hydrophobic interactions rather than hydrogen bonding.

### 2.5. Revealing the Binding Determinants of EGT Using Free Energy Methods

The MM/PBSA analysis supports the results from molecular docking and MD simulations (Table 2). EGT exhibits distinct binding energetics across multiple targets, with AKT1 as the primary regulatory target. TNF and CASP3 act as secondary targets, while IL-6 demonstrates relatively weak direct interaction. These findings suggest that EGT’s pharmacological effects are likely driven predominantly through the AKT1 signaling pathway, while also modulating TNF-mediated inflammation and CASP3-related apoptosis. Together, these interactions provide a theoretical foundation for its neuroprotective role.

### 2.6. Gibbs Free Energy Elucidates the Binding Mechanism of EGT

**Figure 5:**
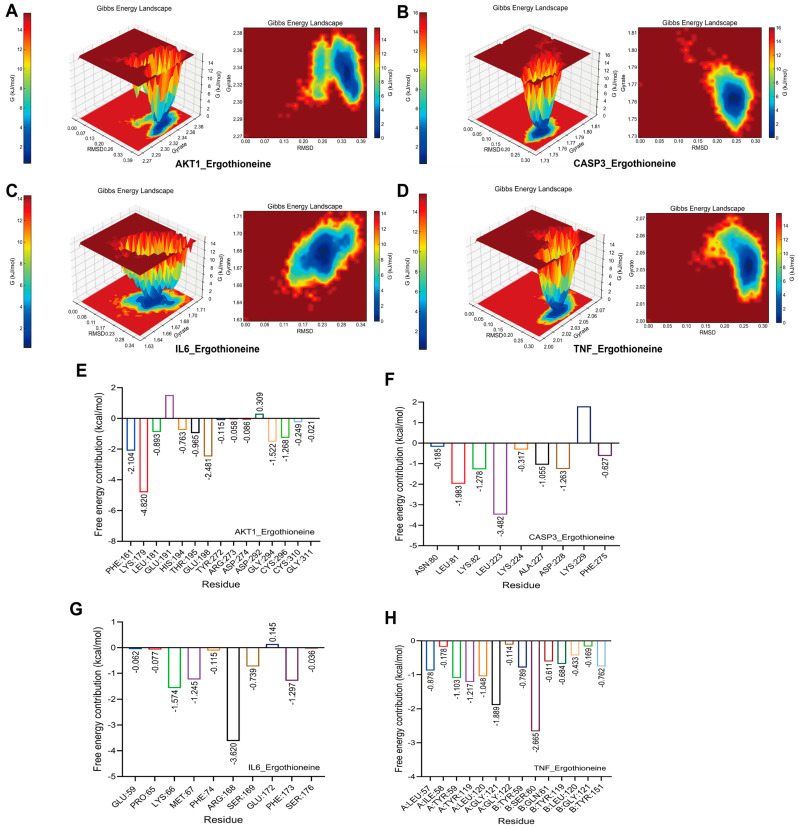
Gibbs free energy analysis and free energy (MM/PBSA) analysis. (**A**) Gibbs free energy diagram of AKT-EGT complex. (**B**) Gibbs free energy diagram of CASP3-EGT complex. (**C**) Gibbs free energy diagram of IL-6-EGT complex. (**D**) Gibbs free energy diagram of TNF-EGT complexes. (**E**) Amino acid decomposition of IL-6-EGT complex. (**F**) Amino acid decomposition of CASP3-EGT complex. (**G**) Amino acid decomposition of IL-6-EGT complex. (**H**) Amino acid decomposition of TNF-EGT complex.

The Gibbs free energy landscapes illustrate how EGT binds to four key proteins (AKT1, CASP3, IL-6, and TNF) (Figure 5A–D), displaying distinct low-energy regions for each. These regions reflect stable and energetically favorable conformation, indicating that EGT interacts reliably with all targets and may influence their signaling and biological activities. This provides insight into how EGT may modulate stress responses, immune regulation, and metabolic processes.

Further analysis of residue-level contributions reveals that specific amino acids play a major role in stabilizing these complexes (Figure 5E–H). For example, LYS179 in AKT1, LEU223 in CASP3, ARG128 in IL-6, and SER60 in TNF showed strongly negative free energy values, confirming their importance in ligand binding.

Analysis of the terminal conformations at 100 ns indicates that ergothioneine exhibits deeper insertion and more pronounced spatial encapsulation within the binding pockets of AKT1 and IL6. In contrast, the binding sites of CASP3 and TNF remain relatively open, resulting in greater solvent exposure of the ligand (Figure S1). Across all systems, the protein backbones remained structurally stable throughout the simulations, with no significant conformational rearrangements observed, suggesting that the complexes had reached dynamic equilibrium.

These differences in binding pocket enclosure imply that ergothioneine may adopt a more stable binding mode with AKT1 and IL6, whereas its interactions with CASP3 and TNF may involve greater conformational flexibility and adaptability. This analysis elucidates the molecular basis of EGT–protein interactions and highlights residues that may be critical for regulating protein activity.

### 2.7. Activity Effects of EGT on BV2 Microglia Cells

**Figure 6:**
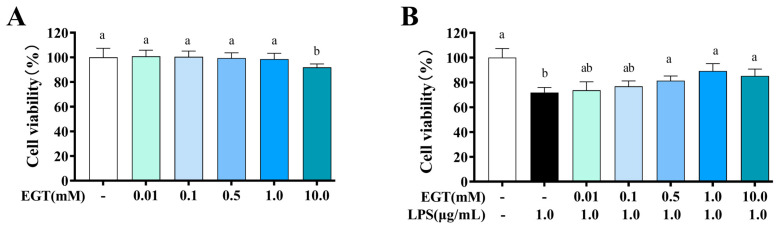
Effect of EGT on BV-2 microglial cell viability. (**A**) BV2 cells were treated with various concentrations of EGT (0.01, 0.1, 0.5, 1.0, and 10 mM) for 24 h. (**B**) BV-2 cells were incubated with EGT (0.01, 0.1, 0.5, 1.0, and 10 mM) and exposed to LPS (1.0 μg/mL) for 24 h. Cell viability was measured by CCK8 assay. Each result is presented as mean ± standard error of mean (SEM) (n = 3). Values with different letters are significantly different (*p* < 0.05).

This study systematically evaluated the activity of EGT on BV2 cells using the CCK-8 method. As shown in Figure 6, within the concentration range of 0.01 to 1.0 mM, the cell viability of the EGT-treated group exhibited no statistically significant difference compared to the control group (CON) (*p* > 0.05), indicating that EGT did not significantly impair cell survival under these concentrations, with cells maintaining high viability and normal growth. However, as the EGT concentration increased further, cell viability decreased significantly (*p* < 0.05), demonstrating a clear concentration-dependent toxic effect. This suggests that EGT at 10 mM exhibited damaging effects on cells. Figure 6B shows that the addition of EGT and LPS resulted in an upward trend in cell viability. Based on these findings regarding cell viability, we ultimately selected three working concentrations of 0.1 mM, 0.5 mM, and 1 mM for subsequent experiments.

### 2.8. EGT Alleviates LPS-Stimulated BV2 Microglia Cell Inflammation

**Figure 7:**
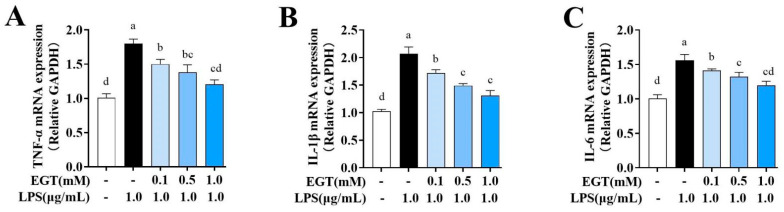
Effect of EGT (0.1, 0.5, 1.0 mM) on mRNA expression of TNF-α (**A**), IL-1β (**B**), and IL-6 (**C**) in LPS (1 μg/mL)-induced BV2 cells. Each result is presented as mean ± standard error of mean (SEM) (n = 3). Values with different letters are significantly different (*p* < 0.05).

To elucidate the molecular mechanisms by which EGT regulates neuroinflammation, we employed real-time quantitative PCR to measure the mRNA expression levels of classical pro-inflammatory factors. Figure 7 shows that the LPS group exhibited significantly elevated expression of TNF-α, IL-6, and IL-1β compared with the CON group. In contrast, EGT treatment markedly reduced the mRNA expression of these cytokines. IL-1β and IL-6 levels were significantly lower in the medium-dose group than in the low-dose group, whereas TNF-α expression did not differ significantly between these two groups. The high-dose group showed an even greater reduction in pro-inflammatory cytokine expression, with levels approaching those of the CON group. These findings indicate that 1.0 mM EGT exerts the strongest anti-inflammatory effect.

### 2.9. EGT Alleviates LPS-Stimulated Neuroinflammation by Inhibiting the NF-κB Signaling Pathway

**Figure 8:**
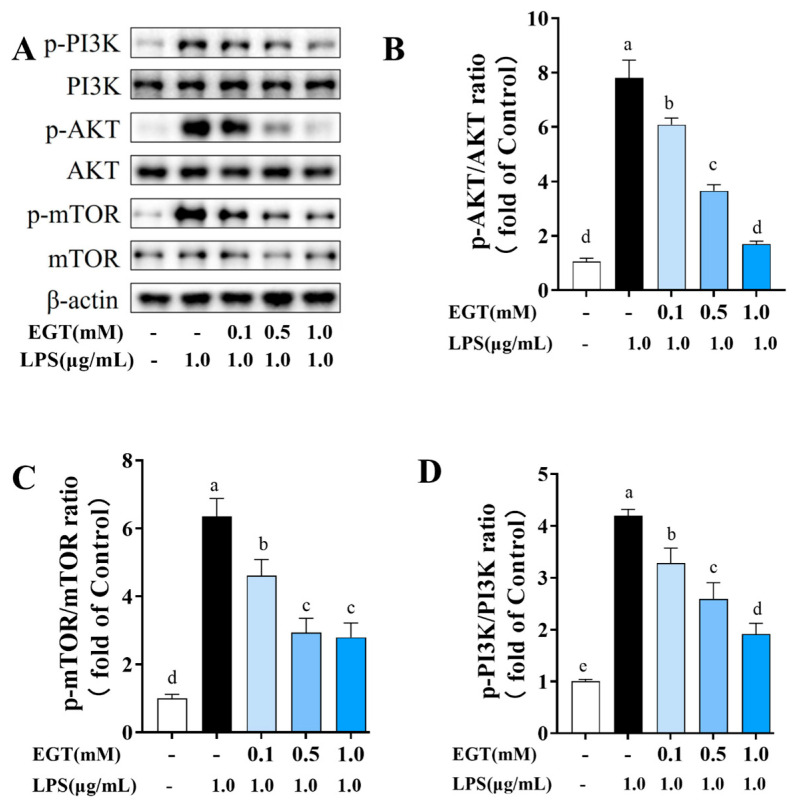
Representative images of Western blotting of EGT (0.1, 0.5, 1.0 mM) influencing protein expression of p-AKT/AKT, p-PI3K/PI3K, and p-mTOR/mTOR. (**A**) Western blotting of p-AKT/AKT, p-PI3K/PI3K, and p-mTOR/mTOR. (**B**) Quantification of ratio of p-AKT/AKT. (**C**) Quantification of ratio of p-mTOR/mTOR. (**D**) Quantification of ratio of p-PI3K/PI3K. Data are presented as mean ± SEM (n = 3). Values with different letters differ significantly (*p* < 0.05).

To elucidate the mechanism by which EGT modulates NF-κB-related protein expression in LPS-stimulated BV2 cells, Western blot analysis was performed to assess key components of the NF-κB signaling pathway (Figure 8). Compared with the CON group, LPS-stimulated BV2 cells exhibited significantly elevated NF-κB p65 phosphorylation levels (*p* < 0.05), indicating activation of the NF-κB signaling pathway. EGT treatment significantly reduced NF-κB p65 phosphorylation, and the inhibitory effect became more pronounced as the concentration increased.

Analysis of upstream NF-κB-related proteins showed that LPS exposure significantly increased TLR4 and MYD88 expression (*p* < 0.05) compared with the CON group, indicating activation of the inflammatory cascade. When EGT was added, levels of TLR4 and MyD88 decreased significantly, with greater reductions observed at higher concentrations. These findings suggest that EGT acts upstream of NF-κB by suppressing TLR4 and MyD88, thereby blocking inflammatory signaling and reducing NF-κB p65 phosphorylation.

### 2.10. EGT Regulates PI3K/AKT-Related Protein Expression in LPS-Stimulated BV2 Cells

**Figure 9:**
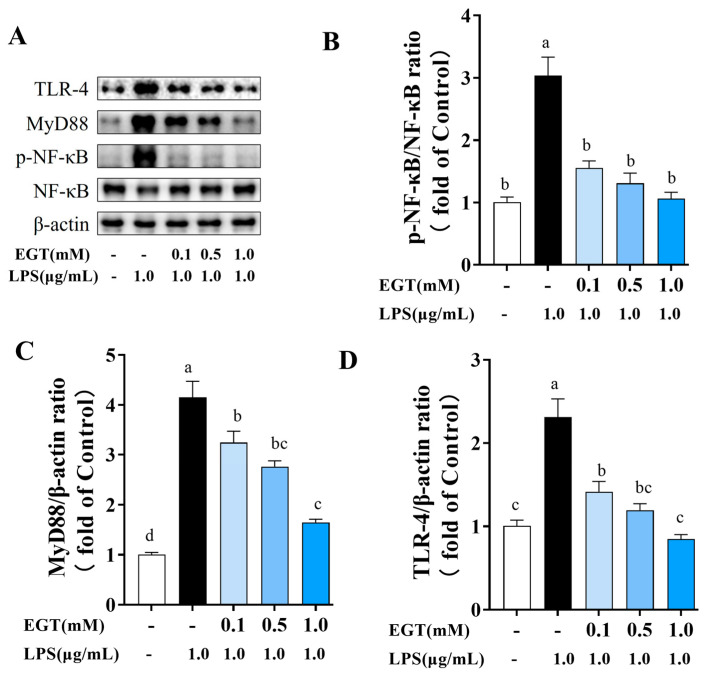
Representative images of Western blotting of EGT (0.1, 0.5, 1.0 mM) influencing protein expression of TLR4, p-NF-κB/NF-κB, and MyD88. (**A**) Western blotting of TLR4, p-NF-κB/NF-κB, and MyD88. (**B**) Quantification of ratio of p-NF-κB/NF-κB. (**C**) Quantification of ratio of MyD88/β-actin. (**D**) Quantification of ratio of TLR4/β-actin. Data are presented as mean ± SEM (n = 3). Values with different letters differ significantly (*p* < 0.05).

Neuropathological inflammation is closely associated with the PI3K/AKT signaling pathway, which regulates cell survival, proliferation, and inflammatory responses [23]. Building on our previous findings, this pathway interacts with others such as HIF-1, Toll-like receptor, TNF, and apoptosis. These observations led us to hypothesize that EGT may modulate inflammation by regulating PI3K/AKT-related proteins. To validate this, we employed Western blotting to detect PI3K, AKT, and their phosphorylated forms in LPS-stimulated BV2 cells before and after EGT treatment. LPS exposure significantly increased PI3K and phosphorylated AKT, indicating pathway activation (Figure 9). Conversely, EGT treatment reduced both PI3K expression and AKT phosphorylation in a dose-dependent manner, indicating that EGT treatment can inhibit this pathway.

Further analysis showed that LPS significantly increased the p-mTOR/mTOR ratio, indicating robust activation of mTOR downstream. EGT treatment significantly reduced this ratio in a concentration-dependent manner, with no detectable difference between the medium and high doses. These results suggest that EGT’s inhibitory effect extends to mTOR signaling. Collectively, EGT appears to provide protective effects by modulating apoptosis and inflammatory responses through coordinated control of multiple targets and pathways, supporting its potential application in neuroinflammation intervention.

## 3. Discussion

Neuroinflammation plays a central role in the progression of neurodegenerative diseases. It is characterized by excessive cytokine release, microglial overactivation, and dysregulated apoptotic processes, which together lead to neuronal injury and cognitive impairment [24]. Therefore, exploring natural compounds that can effectively regulate neuroinflammatory responses holds significant importance. Recent studies suggested that EGT may exert neuroprotective effects by intervening in inflammatory and oxidative stress-related signaling pathways through multi-target mechanisms [25]. However, its specific molecular targets and network regulatory mechanisms in neuroinflammation remain unclear. This study systematically investigated potential EGT targets and mechanisms in neuroinflammation through network pharmacology, molecular docking, PPI analysis, MD simulations, and in vitro functional validation.

Using network pharmacology, 37 overlapping targets associated with EGT regulation of neuroinflammation were identified. Construction of a PPI network further revealed several core targets, including TNF, AKT1, CASP3, IL-6, STAT3, NFKB1, CXCL8, HIF1A, MTOR, and PTGS2. These molecules are closely associated with inflammation and play crucial roles in immune responses and neuroinflammatory processes. Among these, TNF serves as an upstream pro-inflammatory cytokine that initiates and amplifies immune responses. Through TNF-induced activation of the NF-κB signaling pathway, NFKB1 becomes a central transcriptional regulator, promoting the expression of additional inflammatory mediators, including IL-6, CXCL8, and even TNF itself, creating a positive feedback loop that sustains inflammation [26]. IL-6 activates the JAK–STAT pathway, leading to STAT3 phosphorylation and its movement into the nucleus, where it regulates genes involved in immune cell differentiation, survival, and cytokine production [27]. Thus, TNF, NFKB1, IL-6, and STAT3 form a tightly interlinked signaling axis that drives both acute and chronic inflammatory responses. AKT1, a key component of the PI3K–AKT pathway, intersects with NF-κB and STAT3 signaling. Its activation enhances cell survival and modulates cytokine production, with effects that can either amplify or restrain inflammation depending on context [28]. AKT1 also communicates with mTOR, a downstream kinase that integrates metabolic status, immune activation, and autophagy. The AKT–mTOR axis plays a crucial role in determining whether immune cells adopt pro-inflammatory or regulatory phenotypes [29]. At the level of cell fate, CASP3 acts as a pivotal executioner of apoptosis. Its activation not only mediates programmed cell death but also contributes to inflammatory cell death (e.g., pyroptosis-associated caspase cross-talk), which releases intracellular factors that further stimulate TNF and NF-κB signaling. Similarly, inflammatory or hypoxic conditions promote the stabilization of HIF1A, which interacts with NF-κB and mTOR pathways to regulate glycolysis, angiogenesis, and inflammatory cytokine expression. HIF1A thereby links metabolic stress with immune activation. CXCL8, a chemokine transcriptionally regulated by NF-κB and HIF1A, recruits neutrophils and reinforces the inflammatory microenvironment [30], while PTGS2 catalyzes prostaglandin synthesis and contributes to chronic inflammation [31].

KEGG enrichment analysis indicated that EGT modulated multiple inflammatory and immune pathways, including HIF-1, TNF, IL-17, Toll-like receptor, and PD-L1 checkpoint pathways, as well as viral infection-related mechanisms (e.g., Hepatitis B, Measles, Influenza A, EBV). This suggests that EGT acts on central regulatory genes involved in apoptosis, lipid metabolism, and inflammation, highlighting its potential for neuroinflammatory intervention.

To validate computational predictions, molecular docking and MD simulations were performed. The docking results indicate that EGT exhibits favorable binding affinity toward the core targets, forming stable ligand–protein conformations. Although the calculated docking scores of EGT (−4.6 to −6.0 kcal/mol) indicate only moderate binding strength compared with many natural products, this interaction is still biologically meaningful. Unlike high-affinity synthetic inhibitors, the pharmacological effects of EGT are fundamentally dependent on its specific transporter, OCTN1, which enables its accumulation to millimolar concentrations in target tissues. This unique uptake mechanism likely compensates for its sub-micromolar binding affinity, thereby supporting physiologically relevant target engagement [32,33]. This multi-target and moderately high-affinity characteristic indicates that EGT employs a ‘gentle’ regulatory mechanism, prioritizing the maintenance of cellular homeostasis rather than the strong enzymatic inhibition typical of conventional drugs.

The stability and functional consequences of EGT–protein interactions were further assessed using key MD descriptors. Minimal RMSD fluctuations in the EGT–target complexes reflect high conformational stability, suggesting that EGT can maintain prolonged engagement with neuroinflammatory targets under physiological conditions. Consistent Rg values throughout the 100 ns simulation further confirm that EGT binding preserves target compactness and structural integrity [34,35]. Similarly, stable SASA profiles indicate that ligand binding does not expose hydrophobic cores or promote protein destabilization, which aligns with EGT’s established cytoprotective and safety profile [36]. Residue-level flexibility was assessed using RMSF analysis [37]. While most residues exhibited only minor fluctuations (<0.5 nm), reduced RMSF values were observed in the catalytic loop regions of AKT1 and CASP3. This ‘molecular wedge’ effect suggests that EGT may stabilize active-site loops, thereby limiting enzymatic turnover. In addition, the observed hierarchy of hydrogen bond stability (AKT1 > TNF > CASP3) provides insight into target selectivity. These findings are consistent with the MM/PBSA binding free energy results, designating AKT1 as the central pharmacological node. Together, these data suggest a multi-target regulatory framework through which EGT modulates neuroinflammatory pathways.

Additionally, MM/GBSA calculations revealed that the ΔG-bind of EGT to the core targets AKT1 and TNF ranged from −15.2 to −31.92 kcal/mol. These values are comparable to those reported for other bioactive natural compounds in recent studies. For example, Rengasamy et al. identified potent natural inhibitors of AKT1 with MM/GBSA values ranging from −37.26 to −51.02 kcal/mol [38]. Furthermore, Fu et al. demonstrated that indigo and indigo red, screened from Indigofera tinctoria, showed MM/GBSA binding energies of −35.12 and −38.16 kcal/mol for AKT1, respectively [39]. Although EGT displays slightly lower ΔG-bind, its relatively low molecular weight (229.3 daltons) and its ability to accumulate intracellularly via OCTN1 likely make this affinity sufficient to exert meaningful regulatory effects on the AKT signaling pathway [32].

Microglia, as key immune cells in the central nervous system, play a crucial role in initiating and maintaining neuroinflammatory responses [40]. While activated microglia can eliminate pathogens in the nervous system, the inflammatory factors they release may cause secondary damage to neural tissues [41]. LPS, a key component of Gram-negative bacterial cell walls, serves as a classic natural immune stimulant that activates BV2 microglia cells in the nervous system, triggering excessive secretion of inflammatory factors and subsequent neuronal dysfunction [42,43]. Through bioinformatics screening, we identified the targets points and pathways of EGT’s effects on neuroinflammation. To validate these findings, we established an LPS-stimulated BV2 cell inflammatory model to simulate the inflammatory microenvironment of the central nervous system. Previous studies have reported that EGT significantly reverses the upregulation of pro-inflammatory cytokines in hCMEC/D3 human cerebral endothelial cells induced by 7KC [44]. This observation shows similarity to our experimental results, where EGT demonstrated optimal regulatory effects on inflammatory responses at a concentration of 1.0 mM.

EGT has been considered to have potential applications in neurodegenerative diseases. Previous studies have reported that EGT exerts neuroprotective effects by inhibiting oxidative stress and inflammatory responses, thereby reducing neuronal damage and apoptosis [17]. Researchers have confirmed that EGT protects the skin of mice from UV-induced photoaging by modulating cellular damage or death through the PI3K/AKT/Nrf2 signaling pathway [45]. The NF-κB signaling pathway plays a crucial role in the development of neuroinflammation. Under normal physiological conditions, NF-κB remains inactive in the cytoplasm; when stimulated by LPS or other factors, the signaling pathway is activated, prompting its translocation to the nucleus to initiate transcription of inflammation-related genes [46]. The PI3K/AKT signaling pathway plays a complex yet critical role in the regulation of neuroinflammation. Under normal conditions, this pathway helps maintain neural growth, survival, and metabolic balance. When neural tissue is damaged or invaded by pathogens, abnormal activation of the pathway triggers a cascade of events that lead to neuroinflammation. This activation promotes glial cell activity and the release of pro-inflammatory cytokines, further exacerbating the inflammatory responses. On the other hand, it affects neuronal autophagy function: overactivation inhibits autophagy, preventing the clearance of harmful substances and intensifying neuronal damage and death [47]. Experimental results indicate that EGT regulates the expression of neuroinflammation-related genes (mRNA), NF-κB-associated proteins, and PI3K/AKT-related proteins. Overall, EGT regulates the PI3K/AKT signaling pathway and the NF-κB signaling pathway, thereby affecting the release of inflammatory factors in cells and playing a role in the regulation of neuroinflammation.

Comprehensive analysis of network pharmacology reveals that EGT exerts multi-target and multi-pathway effects in regulating neuroinflammation. Molecular docking further demonstrated that EGT has strong binding potential toward key targets, such as AKT1, forming stable ligand–protein complexes. In addition, cellular experiments demonstrate that EGT modulates the PI3K/AKT signaling pathway and the NF-κB signaling pathway, thereby regulating the intracellular release of inflammatory mediators. However, no positive pharmacological control was established in the cell experiments. Therefore, the relative potency of EGT compared to established clinical inhibitors remains to be fully elucidated. Additionally, this study was limited to in vitro experiments and lacked in vivo studies, necessitating the need for further studies to confirm the mechanistic basis and therapeutic efficacy of EGT. Future research should focus on animal experiments of neuroinflammation and include standard inhibitors, such as LY294002 or conventional nonsteroidal anti-inflammatory drugs, as positive controls. Such studies would enable a more rigorous evaluation of the pharmacological potency, therapeutic window, and translational potential of EGT. The present work primarily focused on validating the ‘target–pathway’ axis rather than conducting comprehensive efficacy comparisons. Therefore, subsequent investigations should emphasize systematic efficacy assessments to better define the clinical relevance of EGT in neuroinflammatory conditions.

## 4. Materials and Methods

### 4.1. Target Screening of EGT Active Components

To systematically investigate the potential molecular mechanisms of EGT, we first predicted EGT-associated targets using three databases—SuperPred (https://prediction.charite.de, accessed on 21 July 2025), CTD (https://ctdbase.org/, accessed on 21 July 2025), and SwissTargetPrediction (http://swisstargetprediction.ch/, accessed on 21 July 2025)—to predict component-specific targets, with gene names cross-verified using Uniprot (www.uniprot.org, accessed on 21 July 2025). All predicted targets were combined, deduplicated, and compiled for subsequent analyses.

### 4.2. Construction of Neuroinflammatory Targets

To obtain disease-related targets associated with neuroinflammation, this study conducted searches using ‘Neuroinflammation’ as the keyword in the following databases: GeneCards (https://www.genecards.org/, accessed on 21 July 2025) with a relevance score > 1, and CTD (https://ctdbase.org/, accessed on 21 July 2025) with an inference score > 40. The collected targets were merged and deduplicated, producing a final dataset for downstream analyses.

### 4.3. Construction of Intersection Target–Protein Interaction Network

Predicted EGT targets and neuroinflammation-related targets were compared using Venny 2.1.0 to identify overlapping genes. Protein–protein interaction (PPI) data for intersecting targets were then obtained from the STRING database (https://string-db.org/, accessed on 21 July 2025) and visualized using Cytoscape 3.10.3.

### 4.4. GO Function and KEGG Pathway Enrichment Analysis

GO and KEGG pathway enrichment analyses were performed using Metascape to identify significantly enriched biological processes and pathways [48]. GO analysis classifies functions into three dimensions: MF, molecular activities of gene products; CC, cellular locations of gene product activities; and BP, pathways and broader processes composed of multiple gene product activities. A significance threshold of *p* ≤ 0.05 was applied. Finally, visualization was performed using the MicroBioInfo platform.

### 4.5. Molecular Docking Analysis

This study employed the CB-Dock2 (https://cadd.labshare.cn/cb-dock2/php/index.php, accessed on 24 July 2025) platform to perform molecular docking analysis on the selected ligands and targets [49]. Compounds were imported into the CB-Dock2 platform for structural preparation, including the addition of hydrogen atoms and assignment of partial charges. The crystal structures of the core target proteins were retrieved from the RCSB Protein Data Bank (https://www.rcsb.org/, accessed on 24 July 2025) (AKT1 ID: 3O96, TNF PDB ID: 2AZ5, CASP3 PDB ID: 4P30, and IL6 PDB ID:4CNI). Protein preparation involved the removal of water molecules and the addition of polar hydrogen atoms. For the ligands, hydrogen atoms and Gasteiger charges were assigned. To validate the docking protocol, a redocking procedure was performed using the native ligand for each target protein. The resulting RMSD values were all below 2.0 Å, confirming the reliability of the predicted binding sites and the scoring function. CB-Dock2 computes binding affinities (kcal/mol) and identifies optimal docking poses with the lowest predicted binding energy. Finally, we utilized PLIP and PyMOL v2.6 to analyze the interaction details between the ligand and receptor and to generate 3D interaction maps [50,51].

### 4.6. Molecular Dynamics Simulation Analysis

First, 100 ns MD simulations were performed using Gromacs with the AMBER99SB-ILDN force field [52,53,54]. The initial configuration of the protein–ligand complex was established by converting coordinates from the PDB format to the GRO format. The small-molecule ligand was parameterized using the General Amber Force Field (GAFF) via AmberTools22, with Restrained Electrostatic Potential (RESP) charges accurately derived from Gaussian 16W calculations and integrated into the system topology. The complex was solvated in a cubic box of TIP3P water molecules, with a minimum distance of 1.2 nm (12 Å) between the protein and the box boundaries. System neutrality and physiological ionic strength were achieved by adding Na^+^ and Cl^−^ ions to a final concentration of 0.154 M. Energy minimization was performed using the steepest descent algorithm to remove steric clashes and optimize the initial geometry. A cutoff of 1.0 nm was applied for short-range electrostatic and van der Waals interactions, while long-range electrostatics were treated using the Particle Mesh Ewald (PME) method. All covalent bonds involving hydrogen atoms were constrained using the LINCS algorithm. Temperature and pressure were controlled using the V-rescale thermostat and the Berendsen barostat (1 bar), respectively. Prior to production simulations, the system was equilibrated for 1 ns under both NVT (canonical) and NPT (isothermal–isobaric) ensembles to ensure thermal and pressure stability [55,56,57,58,59,60,61]. Finally, analysis of the MD simulation trajectories included RMSD, RMSF, Rg, SASA, and H-bonds.

### 4.7. Gibbs Free Energy

This study employed the built-in GROMACS v2022.03 utilities g_sham and xpm2txt.py to compute Gibbs free energy from RMSD and Rg values reflecting complex stability and to generate corresponding 2D and 3D free-energy landscapes. These Gibbs free energy maps provide insight into conformational changes induced by protein–ligand interactions [62]. The size and shape of low-energy basins reflect the stability of protein conformations: darker, deeper, and broader blue regions denote more stable, energetically favorable states, whereas fragmented dark regions indicate greater conformational heterogeneity or flexibility. Weak or unstable protein–ligand interactions typically produce multiple irregular, high-energy clusters across the landscape, while strong and stable interactions yield more uniform, smoother energy basins within the potential energy distribution.

### 4.8. Free Energy Analysis

To further evaluate the binding stability of protein–ligand complexes, this study analyzed stable trajectories from 20 ns of simulation using the “gmx_MMPBSA” script (dependent on MMPBSA.py v16.0) [63]. The binding free energy (BFE) of the complexes was calculated through the molecular mechanics–Poisson–Boltzmann surface area (MM/PBSA) method, providing a comprehensive quantitative assessment of the system’s binding stability and affinity [64]. Given the critical importance of BFE in computational biology, it is extensively applied in drug molecule design, protein interaction analysis, and structural optimization. According to the theory described by Kumari et al. [65], the binding free energy is estimated as follows:

$$\Delta G_{\mathrm{bind}}=\Delta E_{\mathrm{mm}}+\Delta G_{\mathrm{solv}}-T\Delta S=\Delta E_{\mathrm{vdW}}+\Delta E_{\mathrm{elec}}+\Delta G_{\mathrm{polar}}+\Delta G_{\mathrm{nonpolar}}-T\Delta S$$

Here, ΔE_mm_ represents the gas-phase molecular mechanics energy, comprising electrostatic interactions (ΔE_elec_) and van der Waals forces (ΔE_vdW_). In the single-trajectory approach, the internal energy term (ΔE__int_) is typically assumed to cancel out and is therefore neglected. ΔG_solv_ denotes the solvation free energy, which is further decomposed into the polar (electrostatic) solvation component (ΔG_polar,_ calculated using the Poisson–Boltzmann equation) and the nonpolar (hydrophobic) solvation component (ΔG_nonpolar_). The nonpolar term is typically estimated based on the SASA. The term −TΔS corresponds to the conformational entropy change upon binding. Due to its high computational cost and limited accuracy, this term is often omitted in comparative studies of structurally similar systems. A more negative ΔG_bind_ serves as a key indicator of protein–ligand binding affinity and stability. A lower BFE value reflects stronger interaction and a more stable complex.

### 4.9. Cell Culture

BV2 microglial cells were obtained from Procell Life Science & Technology Co., Ltd. (Procell, Wuhan, China). Cells were maintained in Dulbecco’s Modified Eagle Medium (DMEM) supplemented with 10% fetal bovine serum, 1% non-essential amino acids, 100 U/mL penicillin, and 100 μg/mL streptomycin, and cultured at 37 °C in a humidified incubator with 5% CO_2_.

### 4.10. Cell Viability Assay

A CCK-8 colorimetric assay was performed to assess the effects of LPS on BV2 cell viability. BV2 cells were seeded into 96-well plates and cultured in DMEM at 37 °C for 24 h. After being washed with PBS, cells were treated with or without LPS (1 µg/mL) in combination with graded concentrations of the test compound (0.01, 0.1, 0.5, 1, and 10 µM), and incubated for 24 h. At each time point, 10 µL of CCK-8 reagent was added to each well, followed by incubation at 37 °C for 4 h. Absorbance was subsequently measured at 490 nm using a Thermo microplate reader (Thermo Fisher Scientific, Waltham, MA, USA), with plates maintained on a constant-temperature shaker prior to reading.

### 4.11. Quantitative Real-Time Polymerase Chain Reaction Analysis

Total RNA was extracted from cell tissues using the TRIzol method, and cDNA was synthesized with the reverse transcriptase Prime Script^TM^ RT (Takara Premix Ex Taq^TM^ II RR036A, Dalian, China), followed by PCR amplification. mRNA expression was detected in real time using the SYBR Green PCR Kit (TaKaRa SYBR^®^ Premix Ex Taq^TM^ II, Dalian, China) and the CFX96TM system (Bio-Rad, Hercules, CA, USA). The standard two-step PCR amplification protocol was 95 °C for 30 s, followed by 40 cycles of 95 °C for 3 s and 60 °C for 30 s. The reaction then entered the melting curve phase. The forward and reverse primers for mice are listed in Table 3. Finally, the relative expression levels of genes were calculated based on the 2^−△△Ct^ value using GAPDH mRNA as the internal reference.

### 4.12. Detection of Intracellular Target Protein Expression

The LPS-stimulated cells were kept in RIPA lysis buffer containing protease inhibitors for 24 h, resuspended gently, and incubated on ice to achieve complete lysis. We centrifuged the lysis buffer in EP tubes at 12,000 rpm for 15 min (4 °C). Supernatants were collected from each cell group and protein concentrations were measured using the BCA kit (Solabo, Beijing, China). We mixed the proteins in calculated proportions, and they were then denatured thoroughly in a boiling water bath. After cooling, the mixture was loaded into polyacrylamide gel lanes for electrophoresis and membrane transfer. The nitrocellulose membrane containing target proteins was blocked with 5% skim milk at room temperature for 1 h and incubated with primary antibodies at 4 °C overnight; then, we washed the protein bands three times with TBST buffer at room temperature. The primary antibodies include β-actin (1:5000; Proteintech; Cat. No. 66009-1-lg), NF-κB p65 (1:1000; Proteintech; Cat. No. 10268-1-AP), p-NF-κB p65 (Ser536) (1:1000; CST; Cat. No. 3033), MyD88 (23230-1-AP), TLR4 (19811-1-AP), PI3 Kinase (PI3K) (1:5000; Proteintech; Cat. No. 60225-1-AP), p-PI3K (1:1000; CST; Cat. No. 4228), AKT (1:2000; Proteintech; Cat. No. 10176-1-AP), p-AKT (Ser473) (1:1000; CST; Cat. No. 8326), mTOR (1:5000; Proteintech; Cat. No. 66888-1-AP), and p-mTOR (Ser2448) (1:1000; CST; Cat. No. 5536). After incubating the protein bands at room temperature for 1 h with the secondary antibody (rabbit anti-HRP-Conjugated Affinipure Goat Anti-Rabbit IgG, 1:4000, Proteintech), the bands were washed three times with TBST buffer. The bands were then placed in a dark chamber for chemiluminescence, compression, and X-ray film exposure and development. Each protein band was tested in triplicate, and the gel imaging system captured images. The grayscale values of each band were calculated using Image J software (NIH Image software, 1.54p).

### 4.13. Data Statistics

Statistical analyses were conducted using SPSS version 26.0, and all data visualizations were generated with GraphPad Prism 9 (GraphPad Software, La Jolla, CA, USA). Comparisons among two or more groups were performed using one-way analysis of variance (ANOVA), followed by least significant difference (LSD) post hoc tests where appropriate. Data are presented as the standard error of the mean (SEM), and differences were considered statistically significant at *p* < 0.05. Each experiment was independently repeated at least three times to ensure reproducibility.

## 5. Conclusions

In summary, this study provides a comprehensive elucidation of the molecular mechanisms by which EGT mitigates neuroinflammation. By integrating network pharmacology with multiscale simulations, we identified AKT1, CASP3, and TNF as key therapeutic targets exhibiting favorable interaction profiles with EGT. Notably, molecular dynamics simulations demonstrated that EGT maintains a stable binding conformation within the active site of AKT1, indicating its high conformational stability under physiological conditions. Experimental validation further confirms that EGT effectively inhibits the release of pro-inflammatory cytokines by precisely regulating the PI3K/AKT/NF-κB signaling pathway. These findings indicate that the therapeutic efficacy of EGT arises from synergistic multi-target regulation rather than inhibition of a single pathway. Overall, this study establishes a robust theoretical and experimental framework for utilizing EGT as a natural food component in neuroprotective agents, bridging the gap between computational target prediction and biological function validation.

## Figures and Tables

**Table 1:** Molecular docking results.

Chemical Compound	Target Protein	PDB ID	Vina Score (kcal/mol)
Ergothioneine	AKT1	3O96	−6.0
	CASP3	4PS0	−4.9
	IL-6	4CNI	−4.6
	TNF	2AZ5	−5.3

**Table 2:** Average BFE (kcal/mol) of EGT to key targets calculated via MM/PBSA method.

Energy Contributions	AKT1	CASP3	IL-6	TNF
ΔE_vdW_	−28.22	−24.18	−18.29	−23.85
ΔE_elec_	−93.52	−19.06	−9.96	−13.74
ΔG_polor_	94.66	31.47	26.11	25.39
ΔG_nonpolor_	−4.83	−3.29	−2.98	−3.00
ΔG_Bind_	−31.92	−15.05	−5.13	−15.20

**Table 3:** Primer sequences used for semi-quantitative RT-PCR analysis.

Gene	Forward Primer	Reverse Primer
IL-1β	TGACGGACCCCAAAAGATGA	TCTCCACAGCCACAATGAGT
IL-6	GAGGATACCACTCCCAACAGACC	AAGTGCATCATCGTTGTTCATACA
TNF-α	CCCTCACACTCAGATCATCTTCT	CTACGACGTGGGCTACAG
GAPDH	TGGAGAAACCTGCCAAGTATGA	TGGAAGAATGGGAGTTGCTGT

## Data Availability

All data generated or analyzed during this study are included in this article.

## Supplementary Materials

The following supporting information can be downloaded at https://www.mdpi.com/article/10.3390/ijms27052179/s1.
