# Supplementary material for: Uncovering the Potential Mechanisms of Ergothioneine in Neuroinflammation Through Network Pharmacology, Molecular Docking, Molecular Dynamics Simulation, and In Vitro Validation
Source: Int J Mol Sci. 2026 Feb 26;27(5):2179. doi: 10.3390/ijms27052179 (PMC12985181; doi:10.3390/ijms27052179)
Supplement: Supplementary file 1 [file ijms-27-02179-s001.zip › ijms-4108605-supplementary.pdf]

Supplementarty Table S1. 37 target for degree

Name	BetweennessCentrality	ClosenessCentrality	Degree
TNF	0.10587766712334185	0.8536585365853658	29
AKT1	0.09630383759152915	0.8536585365853658	29
CASP3	0.08130401948246734	0.8333333333333334	28
IL6	0.07066934136879761	0.813953488372093	27
NFKB1	0.018077837881347522	0.7291666666666666	22
STAT3	0.02554646169450915	0.7142857142857143	22
PTGS2	0.012939171006397897	0.7	21
CXCL8	0.07073095973615005	0.7	21
MTOR	0.02238081850044282	0.7	21
HIF1A	0.019798526156657646	0.7142857142857143	21
FOS	0.0216014051308169	0.673076923076923	20
RELA	0.0028420039344409097	0.625	16
NFE2L2	0.004059903681752421	0.6363636363636364	16
CTSB	0.05650845941009067	0.6363636363636364	16
ACE	0.0087016078884591	0.6363636363636364	16
GPT	0.007921490274431451	0.625	15
NOS2	0.003769204651557593	0.6140350877192983	14
CHUK	0.00257703081232493	0.5932203389830508	13
CDK2	0.0060085852522827315	0.5833333333333334	13
GSR	0.05858288770053477	0.5932203389830508	13
ACHE	0.00941374218685143	0.5833333333333334	12
SLC2A1	0.004858670741023683	0.5932203389830508	12
PTGS1	0.003993148061363583	0.5737704918032787	10
CTSD	0.019696386164502817	0.5555555555555556	9
PIK3R1	1.292824822236587E-4	0.5384615384615384	8
CDK5	0.004119503279167146	0.5223880597014925	7
HSPA1A	0.0022536287242169595	0.5303030303030303	6
CCNE1	0.0011219033067772562	0.5223880597014925	6
MME	3.6937852063902485E-4	0.5303030303030303	6
ACACA	1.4005602240896358E-4	0.5072463768115941	5
P2RX7	0	0.5072463768115941	4
MAP2K2	5.602240896358543E-4	0.5	4
ABCC1	0	0.5	4
NPC1	0	0.39772727272727276	2
GRM4	0	0.4166666666666667	1
CAT	0	0.3763440860215054	1

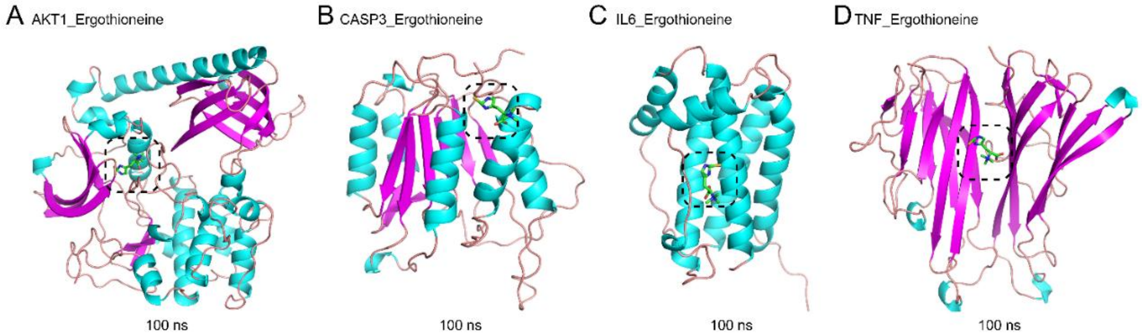

Figure S1. Representative final conformations of protein-ligand complexes obtained from 100 ns molecular dynamics simulations. (A) AKT1 – Ergothioneine complex. (B) CASP3 – Ergothioneine complex. (C) IL6 – Ergothioneine complex. (D) TNF – Ergothioneine complex. The representative structures were extracted from the equilibrated phase of the 100 ns trajectories. Proteins are shown in cartoon representation (α-helices in cyan and β-sheets in magenta), and Ergothioneine is depicted as sticks. Dashed circles indicate the ligand-binding regions. The final conformations illustrate the stabilized binding modes of Ergothioneine within each target protein following dynamic relaxation.
